# The benefits of prosocial power motivation in leadership: Action orientation fosters a win-win

**DOI:** 10.1371/journal.pone.0287394

**Published:** 2023-07-19

**Authors:** Katja M. Friederichs, Karla Waldenmeier, Nicola Baumann

**Affiliations:** Department of Psychology, University of Trier, Trier, Rhineland-Palatinate, Germany; University of Baltistan, PAKISTAN

## Abstract

Power motivation is considered a key component of successful leadership. Based on its dualistic nature, the need for power (*n*Power) can be expressed in a dominant or a prosocial manner. Whereas dominant motivation is associated with antisocial behaviors, prosocial motivation is characterized by more benevolent actions (e.g., helping, guiding). Prosocial enactment of the power motive has been linked to a wide range of beneficial outcomes, yet less has been investigated what determines a prosocial enactment of the power motive. According to Personality Systems Interactions (PSI) theory, action orientation (i.e., the ability to self-regulate affect) promotes prosocial enactment of the implicit power motive and initial findings within student samples verify this assumption. In the present study, we verified the role of action orientation as an antecedent for prosocial power enactment in a leadership sample (*N* = 383). Additionally, we found that leaders personally benefited from a prosocial enactment strategy. Results show that action orientation through prosocial power motivation leads to reduced power-related anxiety and, in turn, to greater leader well-being. The integration of motivation and self-regulation research reveals why leaders enact their power motive in a certain way and helps to understand how to establish a win-win situation for both followers and leaders.

## Introduction

Leadership has long been considered a key driver for organizational success [1]. Today’s leadership requirements are radically changing, however, as modern organizations become increasingly complex, technology accelerates, and the demand for long-term value creation, sustainable growth, and better employee well-being is rising [2]. More than ever, leaders are needed who can empower, relate, and collaborate with their followers, and thus a shift away from traditional, authoritarian, and directive leadership behavior is required [3]. Effective leaders in modern organizations prioritize a positive work culture, team empowerment, and ownership. This creates a motivating and engaging environment where team members feel invested in the organization’s success. Satya Nadella, CEO of Microsoft, and Indra Nooyi, former CEO of PepsiCo, are prime examples of successful leaders who have implemented these practices. Their approaches have led to remarkable business success and revenue growth, showcasing the significant impact of prosocial leadership behavior on organizational achievement.

To understand what motivates leaders to exhibit certain leadership qualities, extensive research has identified the need for power (*n*Power) as an important factor that influences leadership behavior [4–7]. Given the dual nature of *n*Power [8], it can be expressed in a self-serving, aggressive, and assertive manner (i.e., dominant power) but also in an other-serving, benevolent, and supportive way (i.e., prosocial power). Dominant power energizes leadership concerns towards personal gains and status, while prosocial power fuels leaders to empower others and foster the common good [9–11]. Thus, the ability to harness one’s prosocial power becomes increasingly crucial in today’s dynamic business landscape.

Whereas ample evidence highlights the benefits of prosocial power motivation in leaders, such as greater focus on promoting collaboration [9], employee thriving and well-being [12] as well as gender equity [13, see also 14,15], less attention has been given to the factors that contribute to prosocial power enactment. By identifying crucial antecedents, leaders may learn how to leverage the positive aspects of their power motivation and thus create more effective leadership practices. According to Personality Systems Interactions (PSI) theory, high self-regulatory ability (i.e., action orientation) is a decisive predictor for the prosocial enactment of the implicit power motive [16,17]. High self-regulatory ability helps individuals to maintain access to the self and its integrative capacity so that they are able to support rather than ignore the interest of others in their power-related strivings [18–20]. Indeed, prior findings within student samples verified the assumption that action orientation acts as an antecedent of prosocial power motivation [21]. In the present study, we investigated whether this link can also be found in leaders. Further, research has scarcely considered leader´s personal benefits from their leadership behavior [22, see 23 for a review]. Thus, in addition, beneficial effects on leaders themselves were explored, analyzing how leading in a prosocial manner impacts leaders’ power-related anxiety and their well-being.

### Leadership needs power

Leadership above all revolves around power [24–27]. “One cannot be a leader without having power” [25, p.1], as leaders need power to influence, direct, and motivate followers to contribute their efforts towards achieving organizational aspirations [28]. With power at the center of leadership, scholars identified the motivation to obtain power—defined as a strong inner desire to impact others (*n*Power) [29]—as a crucial leader disposition [6,26,30,31]. Individuals high in *n*Power recognize that they contribute to organizational success more effectively by influencing others instead of trying to stand out through their own achievements. Also, they continuously strive for leadership positions and gain satisfaction from their leadership behavior [4,32]. A large body of research has shown that effective and successful leadership is highly correlated with *n*Power [15,33,34]. Further, *n*Power predicts charismatic leadership behavior [35], career progression [6], and advancement into upper managerial roles [36]. Thus, a highly developed *n*Power seems to be vital in leadership.

The need for power, however, has in general a rather poor reputation as it is mostly associated with socially undesired behaviors, such as lack of compassion [37], tendency to harm and dehumanize others [38,39], antisocial decision making [10], or selfishness [40]. Less attention has been given to the benevolent side of the desire to impact others, as it also can energize empowering behavior, such as helping and supporting others [41] as well as mentoring [42], prosocial decision making [10], and greater willingness to forgive others [43]. Moreover, research shows that prosocial power motivation is associated with generativity [44], love for children [45], and greater psychological safety within followers when considered along with supervisor psychological safety [46, see also 47].

The dualistic nature of *n*Power points out that a high need for power may not always turn into egoistic, self-serving, or autocratic leadership, but may also bring forward leaders that aim to benefit others, value relationships with followers, and advance collective interest above personal success and dominance [9,48,49]. Therefore, prosocial power motivated leaders seem to be a valuable asset for organizations and thus it would be beneficial to understand what fosters the benevolent side of *n*Power. The augmented focus on outcome research in the power motivation domain, however, has neglected the question of what determines how individuals enact their *n*Power [30].

### Prosocial power enactment and action orientation

To date, still very little is known about why individuals engage in specific leadership behavior [27] and what determines how *n*Power is enacted [50,51]. Regarding *n*Power as a unitary global construct that is related to toxic and selfish behavior has not contributed to fill this research gap but rather led to contempt power motivation in leadership [52]. In an effort to advocate the importance of power motivation in leadership, James and colleagues highlighted in their recent article that “it is not the power motive that leads to corruption and tyranny, but rather how the power motive is channeled into behavior by other personality factors” [30, p.1]. In line with this, PSI theory suggests that action orientation (i.e., the ability to self-regulate affect) is a decisive predictor for the prosocial enactment of *n*Power [16,17] and initial empirical findings confirm this notion [21].

Action orientation is the ability to self-regulate own emotions and behavior in a context-sensitive way [53–56, for a review see 57]. Action-oriented, relative to state-oriented individuals, show greater psychological functioning in various areas such as professional performance [58], decisiveness and productivity [59,60], and well-being [61]. Several findings show that these benefits are indeed regulated through the self [55,62,63]. PSI theory posits that the prosocial enactment of *n*Power involves the self, and thus is considered an *intrinsic* enactment strategy [64,65]. Intrinsic enactment strategies are driven by positive affect that is not only inherent in the activity itself but mainly results from efficient self-regulation [16,66–68]. In contrast, extrinsic enactment strategies (e.g., dominance) are driven by external incentives (e.g., faces signaling low dominance, [69]) and hence do not rely on the self. Consequently, as action orientation is the ability to regulate emotions through the self, it is considered highly conducive to enact *n*Power in a prosocial way.

Several empirical findings confirm the link between action orientation and intrinsic motive enactment across all social motives (achievement, affiliation, power). For instance, Baumann and Kuhl [18] considered all three motives and showed a significant positive relation of action orientation and self-regulated (e.g., intrinsic) motive enactment. Yet, no relation with incentive driven (e.g., dominant) enactment strategies was observed. In addition, they found that fostering action orientation through intervention leads to greater intrinsic motive enactment (Studies 3–5). Applying different self-regulation trainings, they demonstrated a pre-post increase in self-regulated motive enactment (Study 3), as well as differential treatment effects (Study 4 and 5). Specifically, individuals with low self-regulation ability (i.e., state-oriented individuals) showed more self-regulated motive enactment in the treatment compared to the control conditions (Study 4: humoristic talk; Study 5: no treatment). Moreover, further research shows that action orientation is linked to the intrinsic enactment of the achievement motive (flow [70]), the affiliation motive (intimacy [71]), and the power motive (prosocial guidance [21]).

### The present study

In the present study, we are following up on the results of Baumann and colleagues’ [21] research. Based on the assumptions of PSI theory [17,72] that the self-regulatory ability of action orientation increases the intrinsic, prosocial enactment of the power motive, the researchers examined the relation between action orientation and the prosocial enactment of *n*Power within student samples of aspiring teachers and psychologists. The researchers argued that power motivation is particularly relevant for both professions, as impacting other people by helping, guiding, and transferring knowledge is essential in their daily work. Applying the Operant Motive Test [68,73] they differentiated five enactment strategies within *n*Power (prosocial guidance, status, coping, dominance, and powerlessness) and examined action orientation as an antecedent for the prosocial enactment of *n*Power. Further, they explored personal benefits (explicit power motivation, well-being) of a prosocial enactment strategy. Across both samples (Study 1 and 2) they confirmed their assumption that prosocial enactment of *n*Power is fueled by self-regulation (i.e., action orientation). Furthermore, action orientation was indirectly associated with well-being through prosocial enactment of *n*Power and the explicit power motive.

**Fig 1:**
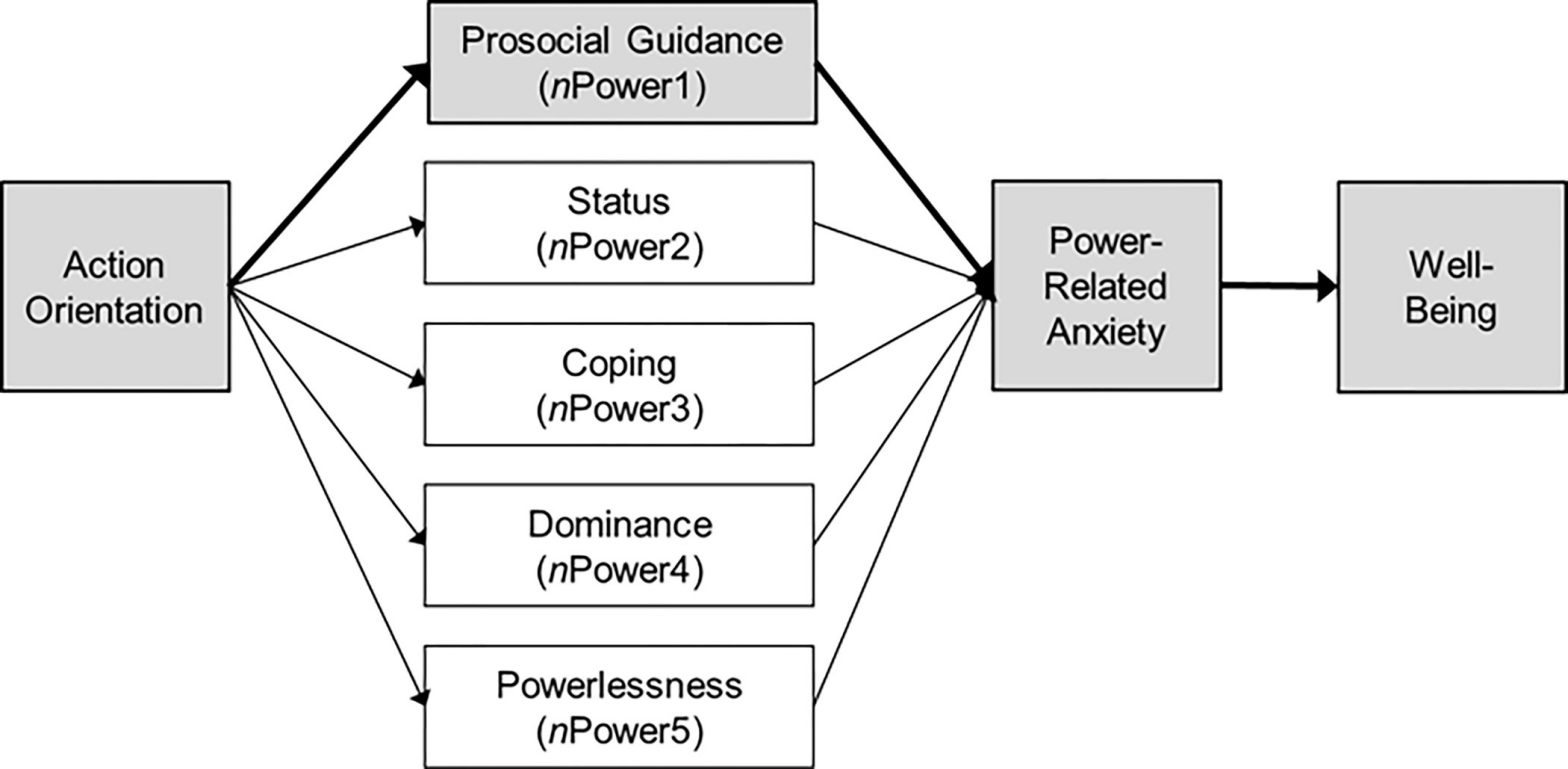
Conceptual model with an indirect path from action orientation through prosocial power enactment (*n*Power1) to power-related anxiety and, in turn, well-being.

As power motivation lies at the center of leadership [26,30]), we examined action orientation as an antecedent of prosocial power motivation in a large leadership sample and expected to replicate the findings of Baumann and colleagues [21]. Our conceptual model is illustrated in Fig 1. Thus, we first tested the relation between action orientation and prosocial enactment of *n*Power and assumed to confirm the link in our sample.

Second, research indicates that the fear of losing power positively correlates with self-serving behavior in leaders [74]. Additionally, power threat may negatively impact leadership behavior even if leaders are generally prosocial oriented [27]. Action orientation, however, has been shown to lead to reduced anxiety in explicit power striving [75,76]. Thus, we analyzed whether action orientation has an indirect effect through the prosocial enactment of *n*Power on power-related anxiety. We assumed an indirect negative effect through implicit prosocial power motivation on power-related anxiety.

Finally, we investigated the impact on leaders’ well-being. To date, a great amount of research has focused on the effect of leadership behaviors on employee’s well-being [e.g., 77], whereas less attention has been placed on leader`s own well-being [22, see 23 for a review]. Based on the insight that action orientation is highly advantageous for well-being [61,75,78] and not only receiving but also giving support is known to be beneficial for well-being [79], we tested whether the indirect path from action orientation through prosocial power enactment on power-related anxiety is associated with leader well-being.

In summary, we tested the following hypotheses: (H1) Action orientation is associated with the prosocial enactment of *n*Power, (H2) action orientation has an indirect negative effect on power-related anxiety through the prosocial enactment of *n*Power, and (H3) action orientation has an indirect negative effect on power-related anxiety through the prosocial enactment of *n*Power and, in turn, well-being.

## Materials and methods

### Participants

Data of *N* = 383 executive leaders (38.40% female) from various companies or organizations were used for the present analysis. Their mean age was 44.08 years (*SD* = 8.57; range 24–72 years). Participants voluntarily filled out of a series of psychological tests, including those relevant for the present research, within the scope of a self-development counseling setting. The assessments were conducted online so that participants were able to complete them on their own computers. Participants provided written informed consent for the use of collected data for research purposes. Ethical approval for this study was not obtained as data was collected by an external institution and provided in an anonymized form. The present data were made available by IMPART (Institute for Motivation and Personality Development: Assessment, Research, and Training; www.impart.de).

### Materials

#### Action orientation

The Action Control Scale (ACS, [56]) was used to assess action orientation. The ACS consists of two subscales assessing decision-related and failure-related dimensions of action orientation with 12 items each. In the present study, decision-related action orientation was relevant (Cronbach’s *α* = .80). An example item is *“When I am facing a big project that has to be done*: *(a) I often spend too long thinking about where I should begin*, *or (b) I don’t have any problems getting started*.*”*. Choice "a" reflects the state-oriented (hesitant) alternative whereas the option "b" indicates the action-oriented (initiative) response. Action-oriented responses were totaled, resulting in scale values from 0 to 12. Hereby, lower scores indicate low action orientation (i.e., state orientation) and higher scores indicate high action orientation.

#### Implicit power motive enactment

**Fig 2:**
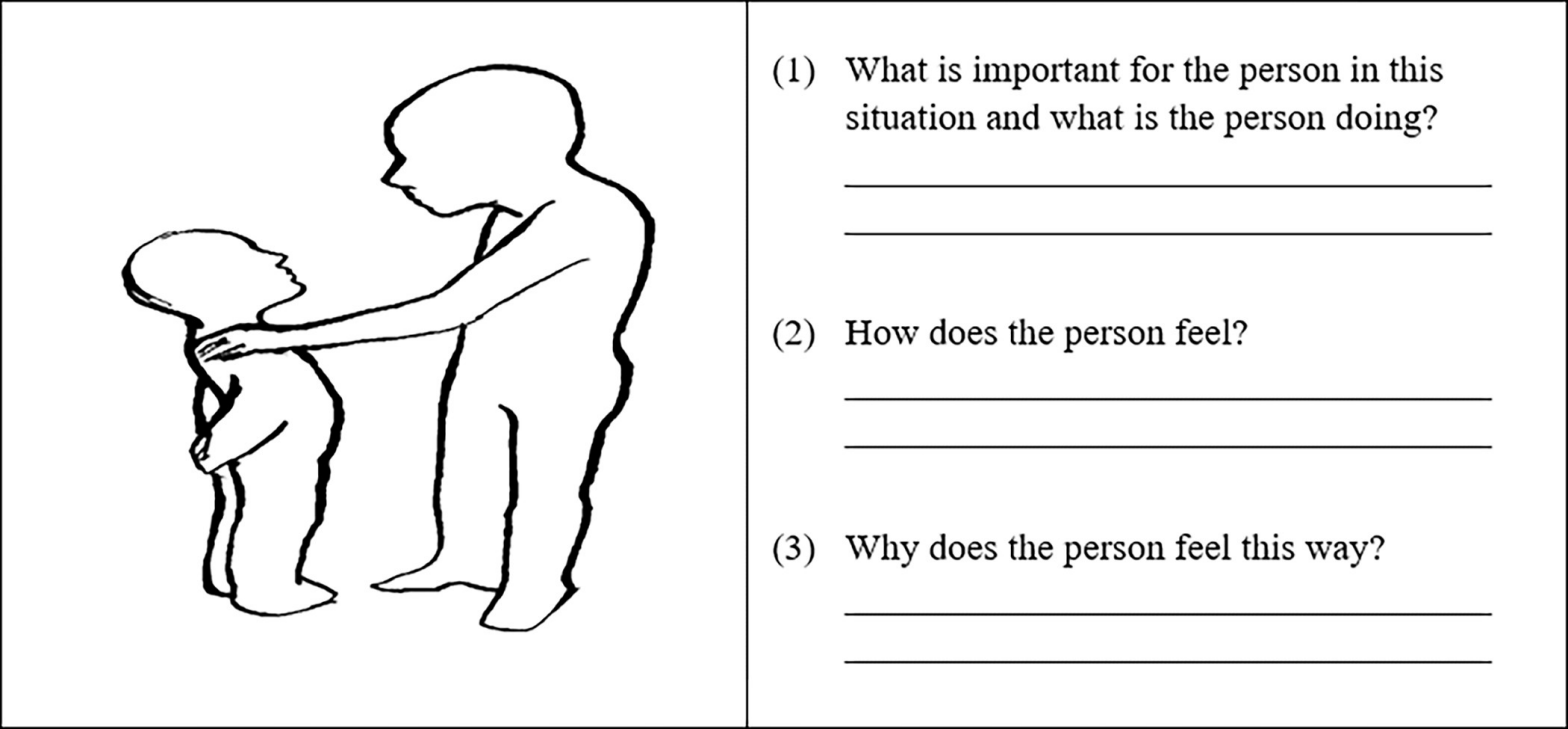
Example picture of the Operant Motive Test (OMT; Kuhl & Scheffer, 1999) that is designed to arouse power motivation.

We applied the Operant Motive Test (OMT, [68]) to measure implicit power motive enactment. The OMT is comprised of fifteen pictures that are designed to either arouse the affiliation, achievement, or power motive. Participants are asked to decide on a main character in each picture, think of a story around that character, and briefly answer three open questions (see Fig 2). The answers are analyzed following a 3-motive x 5-enactment strategies coding procedure. Thereby, each described picture is examined for motive content (i.e., affiliation, achievement, power). A “zero” is coded, if no motive theme can be found. If a motive theme is present, the enactment strategy is determined. To determine the enactment strategy, participants’ answers are screened for approach (*n*Power1-4) or avoidance (*n*Power5) tendencies. Passive avoidance (*n*Power5) is only coded when participants explicitly mention negative affect in their answers and report no active coping or regulation attempts. Approach tendencies (*n*Power1-4) are further screened, differentiating whether they are driven by positive affect (*n*Power1-2) or negative affect (*n*Power3-4). Lastly, descriptions are analyzed whether they involve self-regulation processes (*n*Power1&3, e.g., self-positivity, active coping) or are more external and incentive driven (*n*Power 2&4, e.g., outward focus, goal fixation).

Story Samples: **Prosocial Guidance (*n*Power1)**: *“(1) She wants to help the sitting person*. *(2) Relaxed*, *supportive*, *friendly*. *(3) It’s part of her nature*.*”* **Status (*n*Power2)**: *“(1) She wants to motivate*. *(2) She feels great*. *(3) The person feels she has been confirmed as she acted in accordance with her role/position*.*”* **Coping (*n*Power3)**: *“(1) Performance review*. *Other person has made severe mistakes*. *Empathy and motivation are called for*. *(2) Clear in the leader role*. *Empathetic*. *(3) Regards the mistakes as relative and wants to motivate the person again*.*”* **Dominance (*n*Power4)**: *“(1) She berates the other person*. *(2) assured and superior*. *(3) As she is judging the other person*.*”* **Powerlessness (*n*Power5)**: *“(1) To not get in trouble*. *(2) Anxious*. *(3) Because the person gets scolded*.*”*

It is not of necessity that the main character deliberately experiences the affective source of their motivation and participants do not always explicitly report it in their descriptions. For instance, narratives of rigid and conflict-ridden behavior (e.g., justifying dominant power behavior with role duty) indicate the presence of hidden negative effect that is not being self-regulated. Hence, *n*Power4 is coded. On the other hand, if negative affect is explicitly mentioned but at the same time creative solutions are elaborated (e.g., supports followers to get back on track after providing negative feedback) *n*Power3 is coded. Only if negative affect is explicitly mentioned without an active coping attempt (e.g., feeling powerless in a situation), *n*Power5 is coded. Therefore, negative affect may either be linked to passive avoidance (*n*Power5) or be related to a coping (*n*Power3) or dominant (*n*Power4) enactment strategy. In the same way, positive affect is either linked to prosocial guidance (*n*Power1) or status related enactment (*n*Power2). In contrast to *n*Power2, which is coded when positive affect is provided externally and thus incentives (e.g., status, attention) are assessed in the narratives, *n*Power1 is coded when positive affect seems to flow out of the activity itself (e.g., naturally providing support, when needed), indicating self-regulatory functioning [16,80,81].

#### Power-related anxiety

The Motive Enactment Test (MET, [82]) was used to assess the level of anxiety in explicit power striving (e.g., *“I often feel inferior to people whose behaviour conveys power and superiority”*). The 4 Items (Cronbach’s α = .71) were rated on a 4-point scale (0 = *“not at all“*; 3 = *“completely“*).

#### Well-being

The Complaints Questionnaire (BES, [17]) was used to assess well-being of leaders. It is comprised of 8 Items (Cronbach’s α = .73). Example items are: “*I often struggle to coordinate work and private life”* or “*I felt calm during the last few days*”. Participants rated the extent to which each statement applied to them on a 7-point scale (0 = “not at all”, 6 = “very much”).

#### Procedure

Participants were able to complete the test package via the online platform of IMPART (www.impart.de). They could login from any chosen remote computer with their personalized login information that they were provided in advance. After completion, data was accumulated by IMPART and made available for the present study.

## Results

### Descriptives and correlations

Table 1 offers an overview of the descriptive results and correlations among our study variables. Consistent with our first hypothesis, action orientation was positively correlated with prosocial power motive enactment (*n*Power1). Furthermore, action orientation was negatively correlated with power-related anxiety and positively with well-being. In addition, prosocial guidance (*n*Power1) was negatively correlated with power-related anxiety. Finally, power-related anxiety was negatively correlated with well-being.

**Table 1:** Bivariate correlations (Pearson), means, and standard deviations (*N* = 383).

	(2)	(3)	(4)	(5)	(6)	(7)	(8)	(9)	Scale	*M*	*SD*
(1) Action Orientation	.14**	.03	-.02	-.08	.02	.04	-.40**	.41**	0–12	7.43	3.17
(2) Prosocial Guidance (*n*Power1)		-.04	-.07	-.22**	-.13*	.28**	-.18**	.17**	0–15	1.00	1.06
(3) Status (*n*Power2)			-.12*	-.16**	-.13*	.22**	-.10*	.09	0–15	0.75	0.94
(4) Coping (*n*Power3)				-.15**	-.15**	.42**	-.04**	-.05	0–15	1.31	1.29
(5) Dominance (*n*Power4)					-.11**	.39**	.09	-.18**	0–15	2.56	1.37
(6) Powerlessness (*n*Power5)						.23**	.13*	-.09	0–15	0.96	1.00
(7) Implicit Power Motive (*n*Power)							-.04	-.07	0–15	6.58	1.81
(8) Power-Related Anxiety								-.34**	0–3	0.92	0.61
(9) Well-Being									0–6	4.92	0.53

* *p* < .05 ** *p* < .01.

### Direct and indirect effects on power-related anxiety

To test whether action orientation had an indirect effect through prosocial guidance (*n*Power1) on power-related anxiety, we conducted a mediation analysis with 5,000 bootstrap resamples using the SPSS macro-Model 4 described by Hayes [83,84]. Using this procedure, we computed a point estimate and a 95% confidence interval for the mediation effect.

In the model using enactment strategies of the implicit power motive as dependent variables (see Table 2), action orientation was significantly associated with prosocial guidance (*n*Power1), *R^2^* = .02, *F*(1, 381) = 7.99, *p* = .005. In contrast, action orientation was not associated with any other enactment strategy of the implicit power motive (*n*Power2-5), *F*s < 2.51, p > .11.

**Table 2:** Direct effects of action orientation on the five enactment strategies of the implicit power motive (N = 383).

	*B*	*SE*	*t*	*p*	*Boot LLCI*	*Boot ULCI*
Prosocial Guidance (*n*Power1)						
Action Orientation	.14	.05	2.83	.005	.044	.243
Status (*n*Power2)						
Action Orientation	.03	.05	0.56	.574	-.072	.130
Coping (*n*Power3)						
Action Orientation	-.02	.05	-0.29	.770	-.116	.086
Dominance (*n*Power4)						
Action Orientation	-.08	.05	-1.58	.115	-.181	.020
Powerlessness (*n*Power5)						
Action Orientation	.03	.05	0.45	.655	-.078	.124

*Note*. LLCI and ULCI = Lower and Upper Limit of Confidence Interval.

In the model using the power-related anxiety as a dependent variable (see upper columns of Table 3), there were significant direct effects of action orientation and *n*Power1 indicating that higher action orientation and higher prosocial guidance were associated with lower power-related anxiety. In addition, *n*Power5 was associated with higher power-related anxiety, whereas *n*Power2, *n*Power3, and *n*Power4 were not associated with power-related anxiety.

**Table 3:** Direct and indirect effects of action orientation and the five enactment strategies of the implicit power motive on power-related anxiety (N = 383).

	Power-Related Anxiety					
	*B*	*SE*	*t*	*p*	*Boot LLCI*	*Boot ULCI*
Action Orientation	-.38	.05	-8.08	.000	-.470	-.286
Prosocial Guidance (*n*Power1)	-.11	.05	-2.18	.030	-.204	-.011
Status (*n*Power2)	-.08	.05	-1.69	.093	-.178	.014
Coping (*n*Power3)	-.04	.05	-0.75	.452	-.133	.059
Dominance (*n*Power4)	.04	.05	0.69	.493	-.064	.133
Powerlessness (*n*Power5)	.11	.05	2.30	.022	.016	.209
Indirect Effect of Action Orientation on Power-Related Anxiety through			*b*	*SE*	*Boot LLCI*	*Boot ULCI*
Prosocial Guidance (*n*Power1)			-.015	.008	-.034	-.001
Status (*n*Power2)			-.002	.005	-.014	.008
Coping (*n*Power3)			.001	.004	-.006	.009
Dominance (*n*Power4)			-.003	.005	-.015	.006
Powerlessness (*n*Power5)			.003	.007	-.010	.018

*Note*. LLCI and ULCI = Lower and Upper Limit of Confidence Interval.

The significance of the indirect effect of action orientation through *n*Power1 on power-related anxiety was verified with bootstrapped errors and 95% confidence intervals (CIs). Consistent with our second hypothesis, the indirect effect of action orientation on power-related anxiety through *n*Power1 was significant because the limits of the 95% confidence interval did not include zero (see lower columns of Table 3). No other indirect path was significant. Altogether, the model accounted for approximately 20% of the variance in power-related anxiety, *R^2^* = .20, *F*(6, 376) = 15.31, *p* < .001.

### Direct and indirect effects on well-being

**Fig 3:**
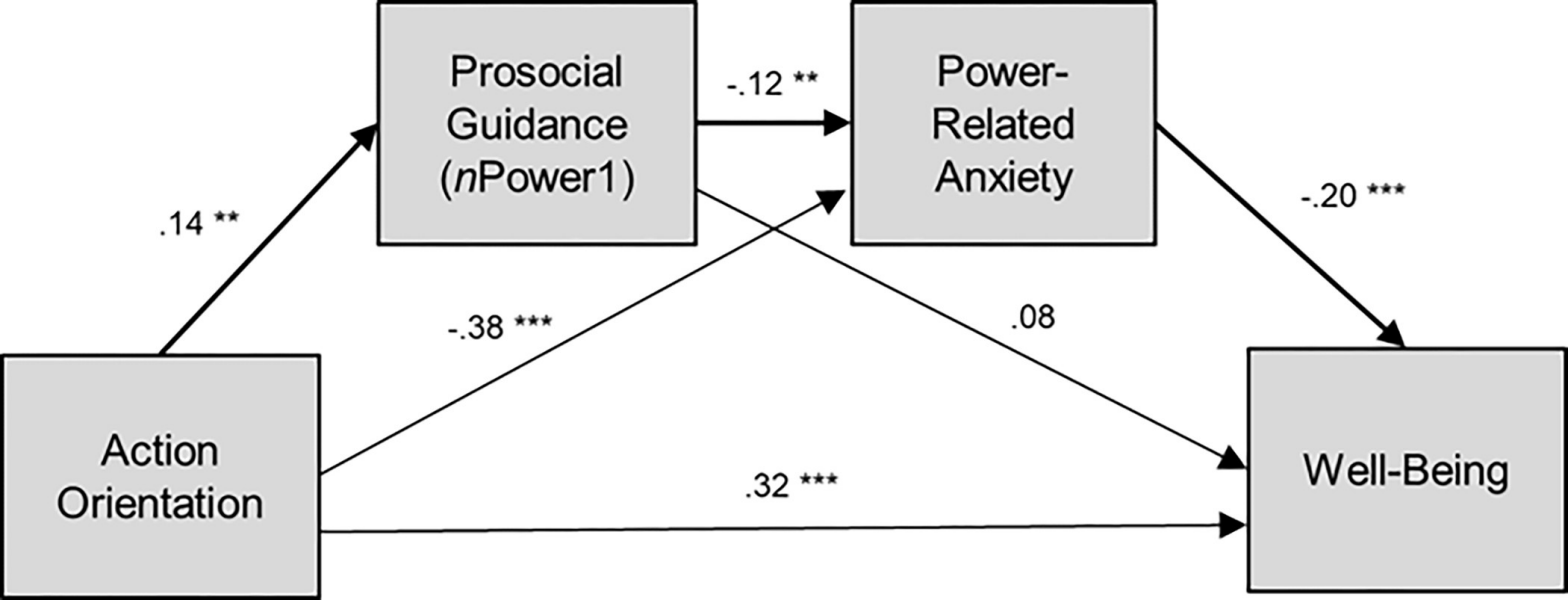
Statistical model with a significant indirect path from action orientation through prosocial power enactment (*n*Power1) and power-related anxiety to well-being.

To test whether there was an indirect effect of action orientation through implicit prosocial power motivation (*n*Power1) and power-related anxiety on well-being, we conducted a mediation analysis with 5,000 bootstrap samples using the SPSS macro-Model 6 [83,84]. With this process, we calculated a point estimate and a 95% confidence interval for the mediation effect. The statistical model and results are illustrated in Fig 3.

As listed in Table 2, action orientation was significantly associated with *n*Power1, *B* = .14, *SE* = .05, *t* = 2.83, *p* = .005 [95% CI: .044, .243]. Consistent with Table 3, when action orientation and *n*Power1 were entered simultaneously to predict power-related anxiety, *n*Power1, *B* = -.12, *SE* = .05, *t* = -2.61, *p* < .01 [-.216, -.030], and action orientation, *B* = -.38, *SE* = .05, *t* = -8.01, *p* < .001 [-.471, -.285], were significantly associated with power-related anxiety. Finally, when action orientation, *n*Power1, and power-related anxiety were entered simultaneously to predict well-being, action orientation and power-related anxiety were significantly associated with well-being whereas *n*Power1 was not (see upper half of Table 4).

**Table 4:** Direct and indirect effects of action orientation, prosocial power enactment (prosocial guidance), and power-related anxiety on well-being.

	*Well-Being*					
	*B*	*SE*	*t*	*p*	*Boot LLCI*	*Boot ULCI*
Action Orientation	.32	.05	6.32	.000	.218	.414
Prosocial Guidance (*n*Power1)	.08	.05	1.82	.070	-.007	.176
Power-Related Anxiety	-.20	.05	-3.94	.000	-.300	-.099
Indirect Effect of Action Orientation on Well-Being through			*b*	*SE*	*Boot LLCI*	*Boot ULCI*
*n*Power1			.012	.008	-.001	.030
Power-Related Anxiety			.075	.022	.035	.122
*n*Power1 and Power-Related Anxiety			.004	.002	.001	.008

*Note*. LLCI and ULCI = Lower and Upper Limit of Confidence Interval.

The significance of the indirect effect of action orientation through *n*Power1 and power-related anxiety on well-being was verified with bootstrapped errors and 95% confidence intervals (CIs). Consistent with our third hypothesis, the indirect effect of action orientation on well-being through prosocial guidance (*n*Power1) and power-related anxiety was significant because the limits of the 95% confidence interval did not include zero (see lower half of Table 4). In addition, the indirect effect of action orientation on well-being through power-related anxiety was significant. Altogether, the mediation model accounted for approximately 21% of the variance in well-being, *R^2^* = .21, *F*(3, 379) = 33.35, *p* < .001.

## Discussion

“A good leader is prosocial” [85, p.283]. Scholars and leadership experts have long called for a new leadership that is characterized by empowering, relational, and collaborative behavior. Early research efforts by McClelland [6] and Winter [11] have identified the need for power as a decisive motivational factor in leadership that can either be expressed in a prosocial or dominant way [8]. Little is known, however, about factors that determine how leaders enact their implicit need for power [30]. In the present research, we took a closer look at personal antecedents and benefits of prosocial power motivation enactment in leaders. Building on the prior research results by Baumann and colleagues [21] who showed that action orientation acts as a predictor of the prosocial enactment of *n*Power, we analyzed this link within a large leadership sample. Our findings confirm that action orientation is linked to implicit prosocial power motivation. Further, we showed that action orientation through prosocial power motivation leads to reduced power-related anxiety and, in turn, to greater leader well-being. The present findings contribute to a better understanding *why* leaders enact their need for power in a certain way: Prosocial leadership is not only a matter of motivation but also of leaders’ self-regulatory ability.

### Theoretical implications

Our findings have several theoretical implications. First, the present research further supports PSI theory’s notion that *intrinsic* motivation depends on unconscious workings of self-regulatory functions [16,65,68,81,86] and complements prior empirical findings demonstrating the link between action orientation and intrinsic motive enactment [18,21,70,71]. Additionally, despite the early conceptualization of implicit assessments [87] and the acknowledged value of implicit processes in leadership, measuring implicit psychological phenomena in organizational settings is still rare (see [88] for a review). On the one hand, this is due to limited access to corporate and non-corporate leader samples. Moreover, as implicit processes operate outside of conscious awareness, they cannot be assessed through self-reports but are assessed with projective measures which are more time consuming for participants and data analysis requires trained experts [89]. This often leads to either only relatively small leader samples in studies or a move back to more accessible student samples when investigating implicit motives. With a relatively large leader sample, we thus contribute to an extended understanding of implicit motives in the leadership context. The conceptual replication of Baumann and colleague’s study [21] with a leadership sample (instead of student sample) further increase confidence in the demonstrated results.

Second, while prior research has emphasized the positive impact of prosocial power motivation on others, there may also be a potential "dark side" to such strivings. If prosocial targets don’t align with company goals, individuals may experience negative outcomes, including work overload, heightened stress [48,90,91], and difficulties in balancing prosocial motivation with actual work tasks [92]. Research conducted by Kibler and colleagues [93] on prosocial motivation within entrepreneurs revealed that it negatively impacts well-being due to the struggle of balancing commercial and prosocial goals. In contrast, however, our research suggests that leaders benefit from their prosocial striving. Despite the daily challenges that leaders face, including balancing competing interests, managing external pressure, and meeting stakeholder demands that may not align with their prosocial strivings, our research reveals that prosocial power enactment contributes positively to their well-being and reduces their power-related anxiety. We propose that self-regulatory ability (i.e., action orientation), may play a critical role in mitigating the potential "dark side" of prosocial power motivation. However, further research is needed to explore this idea in greater depth.

Third, as prosocial leadership behavior decisively impacts the prosperity of organizations [27,94], leaders who naturally strive for making a prosocial impact should be particularly desirable for organizations. However, the desire to impact others is commonly rather discredited as it has been mostly connected to selfish and toxic behavior, and the benevolent manifestation of *n*Power is often overlooked. Concurring with other scholars [e.g., 26,30], the present research points out the value of considering implicit power motivation in leadership, as its prosocial enactment leads to a variety of beneficial outcomes, including, as our results show, for leaders themselves. Moreover, our research goes beyond bringing forward the mere importance of prosocial power motivation in leadership, but also indicates that the benevolent enactment of *n*Power is not only a question of choice but also of ability. Many findings show that action orientation is indeed the *ability* to access and enact motives effectively even under challenging conditions (e.g., high workload, time pressure, stakeholder demands) and without being affected by own emotional states [58,95–97]. The finding that action orientation is an antecedent of prosocial power enactment is therefore good news as self-regulatory ability can be trained and thus a prosocial enactment of the power motive can be fostered.

### Practical implications

Several practical implications can be derived from the present findings. First, our present findings contribute to a currently growing body of research that requests a shift in leadership development from building leadership behavior, skills, and strategies to a greater focus on developing internal attributes that are beneficial to effective leadership [98,99]. The present results further support action orientation as a favorable individual attribute for effective leadership. Research has shown that action orientation develops into advanced old age [100] and can be promoted by intervention [19,101–103]. There are various target-oriented interventions such as mental contrasting [104,105], affective shifting [106], and other established self-regulation methods [e.g., 18,107] that foster action orientation and therefore could promote prosocial enactment within leaders. We hope these findings encourage organizations and leadership consultancies to enhance their focus on nurturing self-regulation abilities within leadership development programs.

The present study goes beyond well-established effects of leadership behavior on employee´s health and well-being [e.g.,^108^]. Contributing to recent efforts in leadership research [23], our study instead highlights the impact on leaders’ own well-being. Paying attention to leader’s well-being in leadership research has far reaching implications. For instance, it supports the identification of beneficial leadership behaviors for both leaders and followers, and thus helps to establish a win-win. Our results indicate that action orientation is a significant enabler for that win-win. Moreover, psycho-symptomatic problems, such as burnout, are quite common among leaders and the prevalence is continuously rising [109]. According to Frieze and Boneva [110], individuals high in power motivation that express it in antisocial or dominant ways (e.g., anger, hostility) are at greater risk to suffer from burnout. In contrast, perceived prosocial impact of own behavior has been shown to act as a protector against burnout [111]. Consequently, we suggest that enacting *n*Power in a prosocial manner may also act as a protective factor notably in power-related occupations, and thus promoting action orientation in leaders may minimize burnout risk among leaders.

Striving for power also means once in power, there is a chance one may lose power again. The possibility of losing power triggers threatening or aversive feelings and people high in *n*Power are presumed to be specifically sensitive towards signals of power constraints [112]. Research shows that leaders under power threat are more likely to act in a self-serving manner [74]—even if they are usually prosocial oriented [27]—and try to sustain power although it may harm the interest of their own group members or organization [112]. For instance, facing a power threat, leaders are more likely to antagonize subordinates against each other to prevent alliances among them [113]. Further, leaders are less inclined to support a power threatening idea and thus have a higher tendency to inhibit knowledge creation within group processes [114]. Action orientation, however, has been shown to lead to reduced anxiety in explicit power striving [75]. Building on this, we demonstrated that action orienation through prosocial power motivation leads to reduced power-related anxiety. This indicates that leaders high in action orientation may experience less power threat concerns and thus show less behaviors that impact followers, colleagues, and organizations in negative ways. Considering these beneficial outcomes, we propose to explore these relations more in future research especially in the leadership context.

### Limitations and future perspectives

The present research is not without limitations that should be addressed in future research. First, we neither collected information about leaders`environments (e.g., company size, sector, non-profit/profit, amount of followers etc.) nor about their position (e.g., CEO, director, team leader, supervisor etc.). Spangler and colleagues [115] suggest that different types of organizations require different types of leadership, implying that there is no gold standard of leadership. Implicit motives are considered rather stable dispositions, whereas their enactment may vary strongly over time in response to context conditions [61,68]. Although, according to our and previous results [21], action-oriented people are more inclined to enact their *n*Power in a prosocial manner, their enactment strategy may vary in different contexts, if required [55]. In contrast to their state-oriented counterparts, this variation is not volatile but based on their self-regulatory ability to adapt to present conditions [56] (Kuhl, 1994). Nevertheless, in future studies, environment and leadership levels should be assessed to capture if action-oriented individuals refer to different enactment strategies specific to a position or environment.

Second, we did not assess followers’ benefits of prosocial leadership but derived them from existing literature [e.g., 14]. Future research should consider assessing specific follower benefits, for example, with 360° assessments when investigating antecedents and benefits of prosocial power motivation enactment. Third, to assess well-being, we asked leaders to report the manifestation of physical and mental complaints, and thus considered the absence of complaints as an indicator of greater well-being. In future research, we suggest verifying the present findings with more established well-being measures, such as the WHO-Five Well-Being Index [116] or the Satisfaction with Life Scale [117].

## Conclusion

Today**`**s leadership requirements in modern organizations are high and more than ever individual leader qualities are in demand that enable and empower followers. Power motivation is highlighted as central in leadership, however, few have focused on its prosocial side. In order to illuminate *why* leaders may enact their power motivation in a more benevolent way, we examined the influence of self-regulation (i.e., action orientation) on power motivation. Our findings yield that it takes action orientation to bring out the benevolent side of *n*Power. Further, a prosocial enactment of the power motive goes beyond increasing the well-being of others, but also boosts personal benefits for leaders themselves and creates a win-win. In conclusion, the present research gives promise to build more great leaders as the ability to empower others can be promoted.
